# Addressing fairness in artificial intelligence for medical imaging

**DOI:** 10.1038/s41467-022-32186-3

**Published:** 2022-08-06

**Authors:** María Agustina Ricci Lara, Rodrigo Echeveste, Enzo Ferrante

**Affiliations:** 1 Health Informatics Department, Hospital Italiano de Buenos Aires, Ciudad Autónoma de Buenos Aires, Argentina; 2 Universidad Tecnológica Nacional, Ciudad Autónoma de Buenos Aires, Argentina; 3 Research Institute for Signals, Systems and Computational Intelligence sinc(i) (FICH-UNL/CONICET), Santa Fe, Argentina

**Keywords:** Machine learning, Image processing

## Abstract

A plethora of work has shown that AI systems can systematically and unfairly be biased against certain populations in multiple scenarios. The field of medical imaging, where AI systems are beginning to be increasingly adopted, is no exception. Here we discuss the meaning of fairness in this area and comment on the potential sources of biases, as well as the strategies available to mitigate them. Finally, we analyze the current state of the field, identifying strengths and highlighting areas of vacancy, challenges and opportunities that lie ahead.

With the exponential growth in the development of artificial intelligence (AI) systems for the analysis of medical images, hospitals and medical centers have started to deploy such tools in clinical practice^1^. These systems are typically powered by a particular type of machine learning (ML) technique known as deep learning (DL). DL methods learn complex data representations by employing multiple layers of processing with different levels of abstraction, which are useful to solve a wide spectrum of tasks. In the context of medical image computing (MIC), examples of such tasks include pathology classification, anatomical segmentation, lesion delineation, image reconstruction, synthesis, registration and super-resolution, among many others^2^. While the number of scientific publications related to DL methods applied to different MIC problems in laboratory conditions has grown exponentially, clinical trials aimed at evaluating medical AI systems have only recently started to gain momentum. In fact, according to the American College of Radiology, to date less than 200 AI medical products related to radiology and other imaging domains have been cleared by the United States Food and Drug Administration^3^.

Recently, the research community of fairness in ML has highlighted that ML systems can be *biased* against certain sub-populations, in the sense that they present disparate performance for different sub-groups defined by protected attributes such as age, race/ethnicity, sex or gender, socioeconomic status, among others^4,5^.

In the field of healthcare, the potential unequal behavior of algorithms towards different population sub-groups could even be considered to go against the principles of bioethics: justice, autonomy, beneficence and non-maleficence^6^. In this context, fostering fairness in MIC becomes essential. However, this is far from being a simple task: ensuring equity in ML deployments requires tackling different and multiple aspects along the whole design, development and implementation pathway. While the implications of fairness in ML for the broad field of healthcare have recently been surveyed and discussed^7^, in this comment we focus on the sub-field of medical imaging. Indeed, when it comes to biases in ML systems that can benefit certain sub-populations in detriment of others, the field of medical imaging is not the exception^8,9^. In what follows we will comment on recent work in the field and highlight valuable unexplored areas of research, discussing potential challenges and available strategies.

## What does it mean for an algorithm to be fair?

Let us start by considering this question in the context of patient sub-groups defined by skin tone or race/ethnicity, where a number of recent articles have compared the performance of MIC systems for suspected ophthalmologic, thoracic and/or cardiac pathologies. For example, when it comes to diagnosing diabetic retinopathy, a severe imbalance in the data used to train a model may result in a strong gap in the diagnostic accuracy (73% vs. 60.5%) for light-skinned vs. dark-skinned subjects^10^. In the same vein, it has been detected that models fed with chest radiography for pathology classification have a higher rate of underdiagnosis for under-served sub-populations, including Black patients^9^, so that the use of these tools could increase the probability of those patients being sent home without receiving the care they need. Lower performance of AI models designed for cardiac MRI segmentation (in terms of Dice coefficient) in this group has also been found^11^, which may result in compound biases if any further diagnostic analysis were required to be done on the automatically delineated silhouette.

**Fig. 1:**
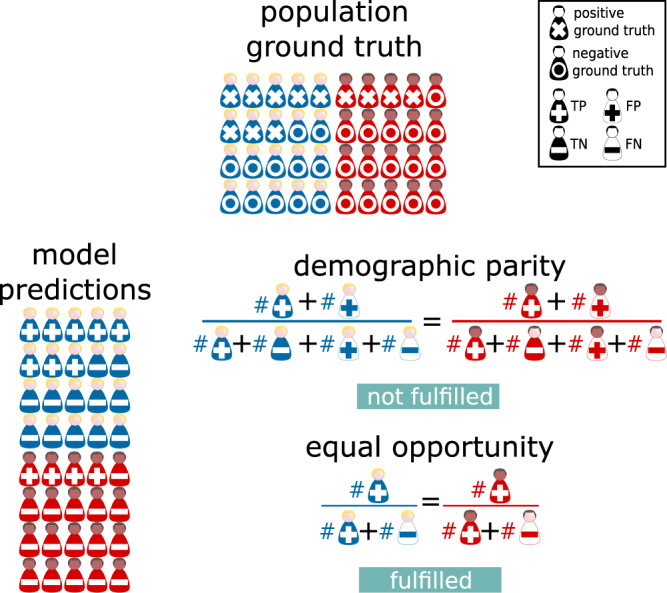
Group-fairness metrics. Here we include a toy-example in the context of disease classification, where two sub-populations characterized by different protected attributes (in red and blue) present different disease prevalence (40% and 20% for blue and red subjects respectively, top row, **x** marks positive cases). A model optimized for discriminative performance was assessed on a test set achieving 100% accuracy (bottom row left side, **+** marks positive predictions). Algorithm fairness was audited according to two common metric choices (bottom row, right side). In this case, as a consequence of the difference in disease frequency, the model would not fulfill the *demographic parity* criterion (bottom row, right side) since the positive prediction rates vary between sub-groups : 40% (8 positive predictions over 20 cases) for the blue sub-group vs. 20% (4 positive predictions over 20 cases) for the red sub-group. On the other hand, the model would fulfill the *equal opportunity* criterion, as true positive rates match for both sub-groups reaching the value of 100%: 8 true positives out of 8 positive ground truth cases for the blue sub-group and 4 true positives out of 4 positive ground truth cases for the red sub-group . FN false negatives, FP false positives, TN true negatives, TP true positives. See legend-box with symbols on the top right corner.

After reading these examples, we immediately and automatically recognize these situations as unfair. However, establishing a criterion to determine whether an algorithm can be called *fair* is actually a thorny issue. In the previous paragraph we have purposely mentioned examples where different metrics were employed in each case. Indeed, the first issue one encounters is that a large number of candidate measures exist. One can for instance evaluate fairness by comparing standard ML performance metrics across different sub-groups, such as accuracy^10,12–16^, or AUC ROC (the area under the receiver operating characteristic curve)^8–10,14–22^, among others. Alternatively, one can choose to employ one of the (no less than ten) different fairness-specific criteria formulated by the community^23^ in order to audit the presence of bias in a given model^16,18^. To complicate matters further, even if one carries out a multi-dimensional study by simultaneously employing multiple metrics^9,10,14–16,20,21,24^, which model to select at the end in a given setting might be no trivial matter and additional information will in general be required. Along these lines, on those occasions when the prevalence of the target condition is different between sub-groups (Fig. 1, top row), special care must be taken in the selection of the fairness definition to be used^25^. For example, the *demographic parity* criterion (Fig. 1, bottom row, right side) which requires equal chances of positive predictions in each group, would here suggest the algorithm is unfair for presenting a higher probability of a positive result for the sub-group with a greater target condition prevalence. This criterion assumes that the prediction of an algorithm is independent of the protected attribute that defines each sub-group, so it may be suitable in settings such as loan eligibility prediction or hiring for job vacancies, but not for disease prediction cases where the prevalence depends on the aforementioned attribute. In these cases, it would be more appropriate to resort to definitions such as the *equal opportunity* criterion (Fig. 1, bottom row, right side), which will compare the equality of true positive rates between sub-groups whose computation is independent of the pre-test probability. Overall, it becomes clear that a one-size-fits-all definition of fairness in MIC will not exist.

## Three reasons behind biased systems: data, models and people

**Fig. 2:**
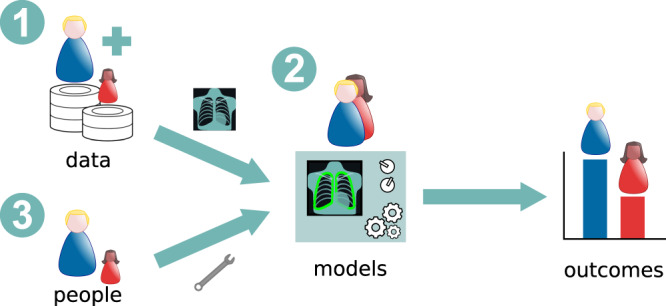
Main potential sources of bias in AI systems for MIC. The data being fed to the system during training (1), design choices for the model (2), and the people who develop those systems (3), may all contribute to biases in AI systems for MIC.

Providing effective solutions to disparities in the outcomes of AI systems starts by identifying which may be their underlying causes (Fig. 2). The lack of diversity and proper representation of the target population in the training databases has been identified as one of the main reasons behind this phenomenon^4^ (Fig. 2①). In the context of MIC, ML systems are trained using big databases of images, usually accompanied by annotations or labels indicating the desired output that we expect from the system (e.g., X-ray images with labels associated with the radiological finding of interest like pneumonia or cardiomegaly). When the demographics of such databases do not match that of the target population, the trained model may be biased, presenting lower performance in the underrepresented groups^11^. Indeed, in chest X-ray pathology classification, only few of the major available datasets in that domain include information about race/ethnicity and, in cases where this information is included, databases tend to be skewed in terms of those attributes^26^.

One point to keep in mind is that a ML system violating one particular definition of fairness should not necessarily be considered biased. In this sense, the selection of appropriate metrics to assess and ensure fairness according to the specific use case is a delicate task that requires careful human intervention. Moreover, such a choice will also be conditioned by the fact that some of these metrics are mutually exclusive^27^, implying that, for example, building a classifier to be simultaneously maximally fair in terms of outcomes, opportunities and calibration will not be feasible most of the time. In addition, other choices related to model design, such as the architecture, loss function, optimizer or even hyper-parameters, may also play a fundamental role in bias amplification or mitigation^28^ (Fig. 2②). The same happens with sampling criteria for database construction. For the above reasons, if decisions are made exclusively by developers, engineers, medical specialists, or data scientists in isolation, or by groups of people who share the same ethnic or social background, there is a risk that their own biases may be unintentionally incorporated into the system based on what they choose to prioritize (Fig. 2③).

Taking a step back, complex structural reasons for bias need also be taken into account. We highlight some of these here (see ref. 7 for an in depth analysis). Unequal treatment of patients, as well as disparate access to the healthcare system due to economic inequalities conspires against investigating certain pathologies in underrepresented populations. Anatomical differences and even variability in the manifestation of diseases across sub-groups can moreover act as confounders. Likewise, many health problems of particular relevance to low income countries are often understudied due to lack of research funding in those countries. Finally, while auditing systems for potential biases, people may unintentionally only search within the possibilities and the reality with which they are familiar.

## Bias mitigation strategies

Several studies in recent years have proposed solutions to mitigate bias and develop fairer algorithms^10,11,14–17,19,20,24^. There are three main stages at which bias mitigation strategies can be adopted^11^: before, during and after training. *Before training*, one would ideally seek to rebalance datasets by collecting more representative data (Fig. 2①). However, in the medical context this is far from trivial as this process requires patients giving consent to their data being used for research purposes as well as the involvement of specialists analyzing each case and providing ground truth labels. Moreover, the low prevalence of certain conditions might hinder finding sufficient examples. In this sense, a compromise solution involves removing features linked to sensitive information, or the use of data resampling strategies. *During training*, several alternatives exist to mitigate model biases (Fig. 2②), such as the use of data augmentation^10,14,19^ and adversarial training^17,20,24^, with the combination of both having even been employed^15^. The use of generative methods as a way to augment the dataset, for instance, has proven effective in reducing the disparity in the diagnostic accuracy of diabetic retinopathy between light-skinned and dark-skinned individuals^10^. On the other hand, adversarial schemes have been shown to reduce biases in skin lesion classification^24^. In this case, adversarial methods intend to increase the performance of a primary model on the target variable while minimizing the ability of a second (adversarial) model to predict the protected attribute from the features learned by the primary model^23^. Finally, *after training*, model outcomes can be post-processed so as to calibrate the predictions across the different sub-groups. These methods focus on the second reason behind biased systems we mentioned before, namely models.

It must be noted, however, that methods designed to improve algorithmic fairness may lead in practice to different outcomes. In the best-case scenario, applying bias mitigation strategies increases the performance of the algorithm for all sub-groups^14^, posing no additional constraints. At the other end of the spectrum, a reduction in the performance for all sub-groups may result from trying to achieve algorithmic fairness^17^. Indeed, interventions to achieve group fairness may result in tensions with the primary goal of the algorithms, requiring a compromise solution. This outcome poses a dilemma in healthcare settings, since it could be interpreted to violate the principles of bioethics, specifically that of non-maleficence. These two extremes are however rare, and a frequent outcome observed in the existing MIC fairness studies analyzed in this article, is performance improvement for the disadvantaged group at the expense of a reduction for another group or groups^11^. This trade-off is also not free of controversies, and once again we find ourselves in a situation where the decision of what is acceptable in a given setting requires careful human consideration. That is why, as discussed in the previous section, diversity is key not only in terms of databases, but also in team composition (Fig. 2③). Hence, considering participatory design practices that explicitly incorporate perspectives from a diverse set of stakeholders^29^ is a fundamental aspect to consider when dealing with algorithmic bias.

## Challenges and outlook for fairness studies in MIC

Even though the field has been steadily growing over the past few years, there are still challenges and open research questions that we believe need to be addressed.

### Areas of vacancy

While this growing trend is highly encouraging, the efforts have been far from even across the landscape of medical specialties and problems being tackled, leaving several areas of vacancy. Firstly, so far algorithmic justice analysis has mostly been carried out in four medical imaging specialties: radiology^8,9,16,18–22^, dermatology^12,13,17,19,24^, ophthalmology^10,14,15^ and cardiology^11^. We believe that this uneven coverage is partly due to the limited availability of MI databases with demographic information on the population (Table 1), something which has been highlighted in several previous studies^8,17^. The absence of this information may be related to the trade-off between data utility and privacy when releasing public databases, in the sense that including sensitive attributes useful for bias audit may go against the privacy of the individuals. To overcome these limitations, the implementation of technical solutions to simultaneously address the demands for data protection and utilization becomes extremely important^30^. Moreover, it must be noted that the subset of sensitive attributes either directly reported or estimated varies from dataset to dataset. The currently most widely reported characteristics are age and sex or gender^16,20,31–38^, followed by skin tone or race/ethnicity^13,16,20,33,34,37,38^, and to a lesser extent socioeconomic characteristics^33,38^. In some cases, where protected attributes are not available, estimates can be computed using image processing methods^12,13,15,19,24^, and eventually manual labeling by professionals can be used^10,13^. These strategies bring with them however an additional level of complexity and subtlety in their implementation which can limit reproducibility and comparison of results across sub-groups.

**Table 1:** Databases commonly used in fairness in MIC studies

Image modality	Database	Access	Sex or gender^a^	Age	Skin tone or race/ethnicity^b^	SES
Chest X-ray	CheXpert^31^	Public	x	x	x	–
	NIH Chest X-Ray^32^	Public	x	x	–	–
	MIMIC Chest X-Ray^33^	Public	x	x	x	x
	Emory University Hospital Chest X-Ray^20^	Private	x	x	x	–
Mammography	Digital Mammographic Imaging Screening Trial (DMIST)^34^	Private	x	x	x	–
	Emory University Hospital Mammography^20^	Private	x	x	x	–
Dermoscopy	ISIC Challenge 2017/18/20^35,36^	Public	x	x	–	–
Dermatological clinical image	Fitzpatrick 17k^13^	Public	–	–	x	–
	SD-198^49^	Public	–	–	–	–
Fundus image	AREDS^37^	Public	x	x	x	–
	Kaggle EyePACS^50^	Public	–	–	–	–
Cardiac MRI	UK Biobank^38^	Public	x	x	x	x
Pulmonary angiography CT	Stanford University Medical Center^16^	Public	x	x	x	–

^a^According to the World Health Organization, *sex* refers to different biological and physiological characteristics of males and females, while *gender* refers to the socially constructed characteristics of women and men such as norms, roles and relationships of and between groups of women and men. Databases tend to report one or the other.

^b^We include both the term *race* and *ethnicity* since the cited studies make use of both denominations. We group analyses across different skin tones in this category as well. *Race* and *ethnicity* are social constructs with complex and dynamic definitions (see ref. 47).

Secondly, important vacancies exist regarding the MIC task to be tackled. The vast majority of studies conducted to date deal with pathology classification tasks^8–10,12–22,24^. The study of fairness in the context of segmentation is however rare^11^, and those of regression, registration, synthesis and super-resolution are rarer still, leaving entire areas to be explored.

### Incorporating fairness audits as common practice in MIC studies

As highlighted by a recent article^17^ which analyzed the common practices when reporting results for diagnostic algorithms in one of the major conferences on MIC, demographics are rarely mentioned, and disaggregated results are infrequently discussed by scientific publications in this domain. This matter is also addressed by the FUTURE-AI Guidelines^39^, which include principles and consensus recommendations for trustworthy AI in medical imaging, and not only focus on fairness but also cover other fundamental dimensions like universality, traceability, usability, robustness and explainability. In that sense, we believe the FUTURE-AI guidelines may constitute a practical tool to improve the publication practices of our community.

### Increasing diversity in database construction

As researchers working in Latin America, we want to stress the importance of widening geographic representation in the building of publicly available MI datasets. It has been acknowledged by several studies that the vast majority of MI databases employed for AI developments originate from high income countries, mostly in Europe and North America^40–42^. This introduces a clear selection bias since the demographics of these countries do not match that of other areas like Africa, Asia or Latin America. This fact, combined with experimental studies suggesting that race/ethnicity imbalance in MI databases may be one of the reasons behind unequal performance^11^, calls for action towards building truly international databases which include patients from low income countries. This issue becomes even more relevant in the light of recent findings which confirm that AI can trivially predict protected attributes from medical images, even in a setting where clinical experts cannot like race/ethnicity in chest X-ray^26^ and ancestry in histologic images^43^. While this fact by itself does not immediately mean that systems will be biased, in combination with a greedy optimization scheme in a setting with strong data imbalance, it may provide a direct vector for the reproduction of pre-existing racial disparities.

In this regard, initiatives such as the *All of Us Research Program*, which invite participants from different sub-groups in the United States to create a more diverse health database, hope to promote and improve biomedical research, as well as medical care^44^. Efforts such as this one, currently focused on an individual country, could be replicated and lay the groundwork for a collaborative enterprise that transcends geographic barriers.

### Rethinking fairness in the context of medical image analysis

For some time now, research on fairness in ML has been carried out in decision-making scenarios such as loan applications, hiring systems, criminal behavior reexamination, among others^23^. However, the field of healthcare in general, and medical imaging in particular, exhibit unique characteristics that require adapting the notion of fairness to this context. Take chest X-ray images for example: particular diagnostic tasks could be easier in one sub-population than the other due to anatomical differences^45^. How to ensure fairness across sub-populations in this case is far from obvious.

Another example is that of existing bias mitigation strategies which may result in reducing model performance for the majority, or even all sub-populations, in exchange for reducing the variance across them. This might be admissible in other contexts, but in the case of healthcare this implies purposely deteriorating the quality of the predictions for a given sub-group, causing ethical and legal problems related to the provision of alternative standards of care for different sub-groups^21^. Moreover, how to define such sub-groups is already an open question: the group-fairness framework, usually applied in problems like loan granting or intended to deal with legal notions of anti-discrimination, reinforces the idea that groups based on pre-specified demographic attributes are well-defined constructs that correspond to a set of homogeneous populations^29^. However, certain attributes like gender identity^46^, are fluid constructs difficult to categorize which require rethinking this framework. Similar issues may arise when using race or ethnicity^47^ as protected attributes to define groups of analysis and evaluate fairness metrics.

While some factors influencing fairness and model performance metrics such as target class imbalance are common to several ML domains, others such as differences in disease prevalence across sub-populations have to be carefully taken into consideration when it comes to MIC. The same holds for the cognitive biases that may be introduced by medical specialists when interpreting and annotating imaging studies^48^. While AI has been postulated as a potential tool to help out in reducing such biases, if not properly addressed, it could also become a mean to amplify and perpetuate them.

Overall there is no denying that the nascent field of fairness in ML studies for MIC still presents important vacancies both in terms of medical specialties and in terms of the types problems being tackled, which will require increased efforts from the community. However, the rapid growth of the field, the development of new guidelines, and the gain of attention reported here, are highly positive and encourage the MIC community to increase its effort to contribute towards delivering a more equitable standard of care.
